# Editorial Correspondence

**Published:** 1872-05

**Authors:** W. F. Westmoreland

**Affiliations:** Philadelphia


### EDITORIAL AND MISCELLANEOUS.

### EDITORIAL CORRESPONDENCE.

PHILADELPHIA, May 10th, 1872.

*Dr. J. P. Logan, Atlanta, Georgia:*

DEAR DOCTOR,—The twenty-third annual meeting of the American Medical Association adjourned this evening, after a session of four days. It was greatly the largest meeting held since the organization of the Association, there being between seven and eight hundred delegates present. It was the opinion of many, that the disturbing elements which, for the past few years, have occupied the greater portion of the time of the Association, to the exclusion of its legitimate objects, would, at the meeting just adjourned, widen the breach, if not destroy the Association, as it had its usefulness for the past two years. All were grateful, however, that these fears were not realized, and that, instead of the wrangling and angry discussions, which were a prominent feature of the two preceding meetings, we had an unusually harmonious session; not that the *inevitable contest* did not present itself, but it was so promptly and by such an overwhelming majority voted out, that it can hardly be said to have interfered with the harmony of the meeting.

As heretofore, the Howard University, Washington, D. C., furnished the material for the disturbance, in one Dr. Rayburn, who claimed admittance as a delegate from this institution, which is a kind of hybrid concern, established by General Howard, of Freedmen's Bureau notoriety. If reports be true, this institution is neither one thing or the other, with male and female teachers, students of both colors and sexes—all blended, whether harmoniously or not, into one organization, constituting the medical department of the afore-mentioned University, and from which emanates, annually, the disturbing element to the Association. From the decided rebuke given by this overwhelming majority, we might be induced to believe that this would forever end the vexed question; but all who for the past few years have watched the machinations of those who have charge of this hydra-headed monster, can but feel that this is not the end, and that annually a head will be presented for decapitation.

Upon the whole, the meeting has been a decided success, and all feel that much good will result in harmonizing the profession, and in giving confidence in the permanency of the Association.

In addition to the sessions of the Association—of which I do not at present propose to speak—we have had much to instruct, interest and amuse. The profession of Philadelphia have spared neither labor or expense in the way of scientific exhibitions, lectures, receptions and excursions, to make our visit in this particular all that could be desired. We have only time, in this hasty letter, to mention some of the more prominent features of these entertainments.

On Tuesday evening, the Biological and Microscopiccal Sections of the Academy of Natural Sciences gave a microscopical exhibition, at which they had one hundred microscopes mounted, illustrating a variety of subjects. The exhibition was novel, instructive and amusing.

The Alumni of Jefferson Medical College held their annual meeting on Wednesday, at one o'clock, and after the conclusion of the exercises, repaired to the residence of Dr. S. D. Gross, where he had in waiting a sumptuous repast.

The Alumni of the University held their meeting on Thursday, at the same hour, and I learn was largely attended.

On Wednesday evening, in addition to lectures at the University and Jefferson Medical College, of an exceedingly interesting character—by Drs. Rogers, Noyes, Cohen, and others—Prof. Hodge and Dr. Wm. H. Pancoast gave magnificent entertainments, where all appeared to vie with each other in adding to the enjoyment of the occasion.

On Thursday, an excursion to the Navy-Yard in the afternoon, and a grand reception in the evening, at the residence of Mr. Scott, the great railroad man of the period, were the prominent features of the day's festivities.

On Friday, an excursion to Fairmount Park, with its attendant lunches, refreshments, etc., and a reception in the evening at Dr. Wistar's, concluded the festivities.

I have made arrangements with the accommodating Permanent Secretary, Dr. Atkinson, to have forwarded an abstract from the official proceedings in time for the June number.*

Yours, truly, W. F. WESTMORELAND.

*The Permanent Secretary, Dr. W. B. Atkinson, Philadelphia, 1400 Pine street, will shortly issue the full proceedings (not including the papers) in pamphlet form, which will be furnished to those sending for it, at fifty cents.

